# Effector T cell subclasses associate with tumor burden in neurofibromatosis type 1 patients

**DOI:** 10.1007/s00262-016-1871-0

**Published:** 2016-07-23

**Authors:** Said Farschtschi, Su-Jin Park, Birgit Sawitzki, Su-Jun Oh, Lan Kluwe, Victor F. Mautner, Andreas Kurtz

**Affiliations:** 1 Neurologische Klinik, Universitätsklinikum Hamburg-Eppendorf, Martinistrasse 52, 20246 Hamburg, Germany; 2 Berlin-Brandenburg Center for Regenerative Therapies, Charité - Universitätsmedizin Berlin, Augustenburger Platz 1, 13353 Berlin, Germany; 3 Institute for Clinical Immunology, Charité - Universitätsmedizin Berlin, Augustenburger Platz 1, 13353 Berlin, Germany; 4 College of Veterinary Medicine, Seoul National University, 599 Gwanangno, Gwanak-gu, Seoul, 151-742 Republic of Korea

**Keywords:** Neurofibromatosis type 1, Immune phenotype, T cell, Plexiform neurofibroma, Whole-body MRI

## Abstract

Neurofibromatosis type 1 (NF1) is a hereditary tumor syndrome caused by mutations of the *NF1* gene and resulting dysregulation of the Ras-pathway. In addition to peripheral nerve tumors, affected tissues include the musculoskeletal and cardiovascular system. The immune system has recently been suggested as a possible modulator NF1-related phenotypes. Therefore, we determined the immune phenotype in NF1 patients and investigated its relationship with the phenotypic severity of NF1-related tumor manifestations. We quantified global leukocytes and lymphocyte subpopulations of peripheral blood from 37 NF1 patients and 21 healthy controls by flow cytometry. To associate immune phenotype with tumor phenotype, all NF1 patients underwent whole-body magnetic resonance imaging and total internal tumor volume was calculated. The immunophenotypes were compared among four NF1 groups with different total internal tumor burdens and between NF1 patients and non-NF1 subjects. We found that NF1 patients show a generalized lymphopenia. Closer analysis revealed that the CD8^+^/CD27^−^ and CD8^+^/CD57^+^ effector T cell fractions strongly increase in NF1 patients with low tumor load and decrease to levels below control in patients with high tumor load. Moreover, increased production of IL2, IFN-γ and TNF-α was found in T cells of NF1 patients upon phorbol-12-myristate acetate (PMA) stimulation compared to healthy controls. The data indicate that decreasing CD8^+^/CD57^+^ and CD27^−^ T cell fractions correspond to increasing tumor load in NF1 patients, potentially making these populations useful marker for internal tumor burden.

## Introduction

Neurofibromatosis type 1 (NF1) is a rare autosomal dominant tumor syndrome, with an estimated incidence at birth of 1 in 3000 [1] and complete penetrance. NF1 is caused by heterozygous mutations in the *NF1* gene, which codes for the tumor suppressor protein neurofibromin acting as a Ras-negative regulator via its Ras-guanosine triphosphate (GTP) activating domain (GAP)-domain. Mutations of *NF1* lead to increased Ras-activity in affected cells [2, 3]. NF1 patients are prone to develop multiple phenotypic manifestations involving the skin, peripheral and central nervous system, musculoskeletal system and cardiovascular system. Multiple neurofibromas are the hallmark of the disease and affect almost all patients. Although benign, neurofibromas can cause severe secondary symptoms such as disfigurement and pain. Musculoskeletal phenotypes of NF1 include scoliosis, long-bone pseudarthrosis and generalized osteoporosis; cardiovascular symptoms may result from involvement of the heart itself or of cerebral, coronary, renal or other arteries. In addition, cognitive impairment and other neuropsychological problems are common [4]. Expressivity of the NF1 phenotype is highly variable and risk assessment problematic due to lack of predictive markers.

There is increasing evidence from animal models and clinical observations, supporting an association of immune phenotypes with several NF1 phenotypes. Abundant mast cells were recognized as a frequent histopathological feature of benign neurofibromas more than 35 years ago [5], an observation that has been repeatedly confirmed [6–8]. Although suggested that these inflammatory cells may be involved in the pathogenesis of neurofibromas in patients with NF1 [9], it was the observation that myeloid malignancies, which occur much more often than expected in children with NF1, frequently exhibit loss of activity of both *NF1* alleles in people with NF1 [10, 11] that delivered evidence for the functional importance of neurofibromin in myeloid cells. The subsequent demonstration that development of neurofibromas in mouse models required *Nf1* haploinsufficiency in myeloid cells of the tumor microenvironment [12–15] established the central role of these bone marrow-derived cells in the pathogenesis of NF1.

Likewise, the hematopoietic microenvironment has been shown to be involved in development of both NF1 vascular disease and NF1 bone disease. For example, NF1-associated vascular disease was associated with a striking increase in vascular inflammation and vascular monocytes in humans [16]. Experiments in a murine model have demonstrated that vascular neointima formation is enhanced by myeloid cells lacking *Nf1* [17]. Similarly, deficient fracture healing and other skeletal manifestations of NF1 have been shown to require the presence of *Nf1* haploinsufficiency in bone marrow mononuclear cells in a mouse model [18]. In addition, an increase in brain microglia in NF1 patients has been reported [19]. The available data substantiate increased circulating monocyte numbers and higher migratory and proliferative capacities of macrophages in NF1 patients and in *NF1*-deficient mice [8, 12–17]. It was furthermore demonstrated that NF1-deficient mice display aberrations in T cell development and function, with reduced proliferative responsiveness to interleukin2 and modulated numbers of naïve T cells [20, 21]. Of note, HLA-DR was found upregulated in *NF1*^−*/*−^ Schwann cells, while it is almost undetectable in normal Schwann cells [22]. Moreover, aberrant plasma cytokine levels in NF1 patients were found in several studies, indicating generalized immunological imbalances with a tendency to develop a systemic inflammatory environment [23–26].

In summary, there is growing evidence for an altered hematopoietic phenotype due to *NF1* haploinsufficiency, which may play a role in the modulation of one or several of the multiple NF1 phenotypes such as vascular disease, idiopathic hypertension, fracture healing and other skeletal manifestations as well as tumorigenesis. However, association of immunological and clinical phenotypes has not yet been systematically assessed with quantifiable clinical data in a larger number of NF1 patients. In particular, we were interested in assessing the lymphocyte fraction of the adaptive hematopoietic cell repertoire and its association with the highly variable tumor phenotype in NF1 patients. Therefore, we profiled the cellular immune phenotype of NF1 patients and correlated it with the quantified internal tumor volume of plexiform neurofibromas (PNF) obtained by magnetic resonance imaging (MRI) of the whole body.

## Materials and methods

### Patients and samples

Adults and children with NF1 were recruited after informed consent at the University Hospital Hamburg-Eppendorf. Blood samples from adult healthy subjects were obtained at Charité University Medicine Berlin after informed consent. Altogether, 37 adult NF1 patients, 9 NF1 patients under 18 years and 21 healthy subjects were included. All NF1 patients were clinically diagnosed according to published guidelines and criteria [27]. Patients with microdeletions involving the NF1 gene, astrocytoma, optical pathway glioma, spinal tumors and schwannoma were excluded from the study. The study was approved by the internal review board (Ethics Committee of the Ärztekammer Hamburg number OB-089/04).

For flow cytometry, the following groups were used: healthy control subjects (*n* = 21, age range 18–60 years, mean 36 years, 11 male, 10 female) and NF1 patients (*n* = 37, only subjects over 20 years of age were included, range 20–66 years, mean 36 years, 18 male, 19 female). Correlation with tumor load was according to the following groups: no internal PNF tumors (*n* = 6), low tumor load (tumor volume <99 cm^3^; *n* = 11), medium tumor load (tumor volume 100–500 cm^3^; *n* = 9), high tumor load (tumor volume >500 cm^3^; *n* = 11).

### Blood differential test

May–Grunwald–Giemsa-stained-whole-blood smears were microscopically examined independently by two persons using standard routine protocols in a double-blinded fashion. Standard reference ranges of leukocyte populations were used for comparison [28].

### Flow cytometry

FACS of PBMC was used to analyze the ratio of different lymphocyte T cell populations. PBMC were prepared from 15 ml to 20 ml venous blood samples collected in sodium citrate as anticoagulant by density gradient in Leucosep™ tubes and Ficoll-Paque Plus solution (Amersham Biosciences) according to manufacturer’s instructions. PBMC were washed with sterile PBS (Biochrom), re-suspended, counted and cryopreserved with 10 % DMSO in FBS (5x10E6 cells/vial) and stored in liquid nitrogen. Flow cytometric analyses were conducted with FITC-, PE-, PE-cyanin 7 (PE-Cy7)-, PerCP-, electron coupled dye (ECD)-, Alexa Fluor^®^ 647-, Alexa Fluor 700-, Horizon™ V450-, Horizon™ V500-, allophycocyanin (APC)-, APC cyanin 7 (APC-Cy7)-, Qdot^®^ 655 nanocrystal- and allophycocyanin-conjugated antibodies. The following antibodies were used: Anti-human CD8 (Life Technologies), CD336 (Becton–Dickinson), CD314 (Beckman Coulter), CD3 (Life Technologies), CD335 (R&D Systems), CD62L (Beckman Coulter), CD56 (Becton–Dickinson), CD4 (Becton–Dickinson), CD16 (Becton–Dickinson), CD25 (Becton–Dickinson), CD127(eBioscience), HLA-DR (Becton–Dickinson), CD57 (Miltenyi Biotec), CD45RO (Becton–Dickinson), CD27 (Life Technologies). Live/dead-discrimination staining dye (Life Technologies) was added to exclude dead cells. After thawing, cell number and viability was microscopically assessed with trypan blue (Sigma) before surface staining. Cells were incubated for 30 min at 2–8 °C and washed afterward. Cells were measured with a LSR-II flow cytometer (Becton–Dickinson), and FACS data were analyzed using FlowJo software (Tree Star Inc.). The following panels were used: T cells (T_H_/T_cyt_) CD3^+^ CD4/CD8; naïve T cells CD4^+^/CD45RO^−^; effector/central memory T cells CD45RO/CD62L; naturally occurring regulatory T cells CD25^+^/CD127^lo^; late activation marker memory T cells CD4/CD8/CD57^+^; late differentiation T cells CD27^−^; early activation marker expressing T cells HLA-DR; natural killer (NK) cells CD3/CD56; cytotoxic NK cells CD16^+^/CD56^dim^; regulatory NK cells CD16^+^CD56^bright^.

### Tumor quantification

Whole-body MRI was performed in NF1 patients to quantify total internal tumor volume. MRI and calculation of total tumor volume were performed in analogy to previous studies [29, 30]. All patients underwent an identical scan protocol with the same parameters for slice thickness, gap, orientation, field of view, imaging matrix, image resolution, echo time, repetition time and inversion time (Siemens Avanto 1.5T). The subjects were imaged in a supine position from head to knee in four steps (head, thorax, abdomen and legs) in accordance with the maximum range of table movement. Slice thickness is 5–10 mm without skips between slices. MRI analysis was performed by an experienced radiologist and a physician trained in image analysis of NF1 associated tumors. The analysis was carried out in a blinded manner. Tumor segmentation and volumetry were performed semiautomatically with the heuristics-based software MEDx (V 3.44) using fat-suppressed T2-short-Ti inversion-recovery (STIR) sequences. Differences in signal intensity of neurofibroma tissue and surrounding tissue were used to define tumor margins on axial slices. The method used for this automated volumetric analysis is sensitive (it detects volume changes as small as 10 %), reproducible (coefficient of variation 0.6–5.6 %) and produces results similar to manual tumor tracings (*R* = 0.999).

Whole-body cutaneous and subcutaneous neurofibroma burden were independently examined by two observers and averages used. Tumor number was estimated within set ranges of (0, 1–10, 11–50, 51–100, 101–500, 500+, 1000+).

### Intracellular cytokine staining

Cytokine production was measured by CD4 and CD8 cells from age- and gender-matched NF1 patients and healthy controls after stimulation. PBMC were stimulated for 4 h with PMA/ionomycin (Sigma), and cytokine secretion was blocked with brefeldin A (BD Biosciences). Cell fixation and permeabilization were carried out with the BD Fixation/Permeabilization Solution kit. Antibodies directed against IL-2, TNF-α and IFN-γ (BD Biosciences) were used to label intracellular cytokines for FACS analysis with a LSR-II flow cytometer (Becton–Dickinson), and data were analyzed using FlowJo software (Tree Star Inc.).

### Statistical analysis

To verify all data for normal distribution, the Kolmogorov–Smirnov test was performed. Stratified patient groups were compared using the Mann–Whitney U test for continuous nonparametric variables. For significance testing, the nonparametric Mann–Whitney U test followed by Bonferroni correction was performed. Two-site tests were used for all analyses. *p* values <0.05 were considered significant. Statistical analysis was performed using SPSS version 18 software (SPSS, Inc., IL, USA) and GraphPad Prism software 5.0 (GraphPad Software Inc., CA, USA).

## Results and discussion

**Fig. 1:**
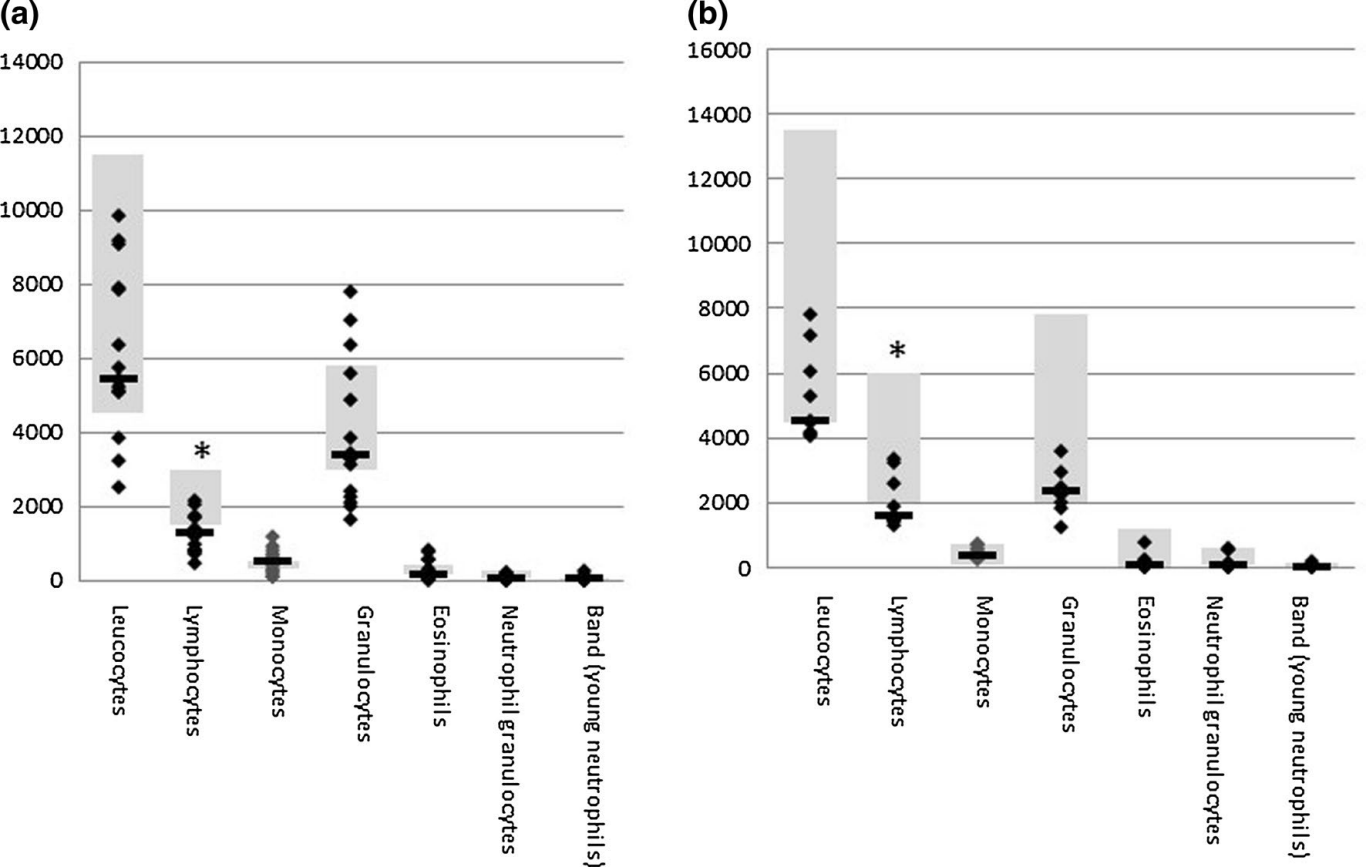
Blood differential test of NF1 patients. Reference ranges of leukocyte populations in samples of healthy subjects are indicated by *gray boxes*. **a** Adults with NF1 (*n* = 15, age range 18–68, median 39 years), **b** NF1 patients under 18 years (*n* = 9, age range 4–17, median 13 years). The lymphocyte levels are below the normal range for both groups (*p* < 0.05)

**Fig. 2:**
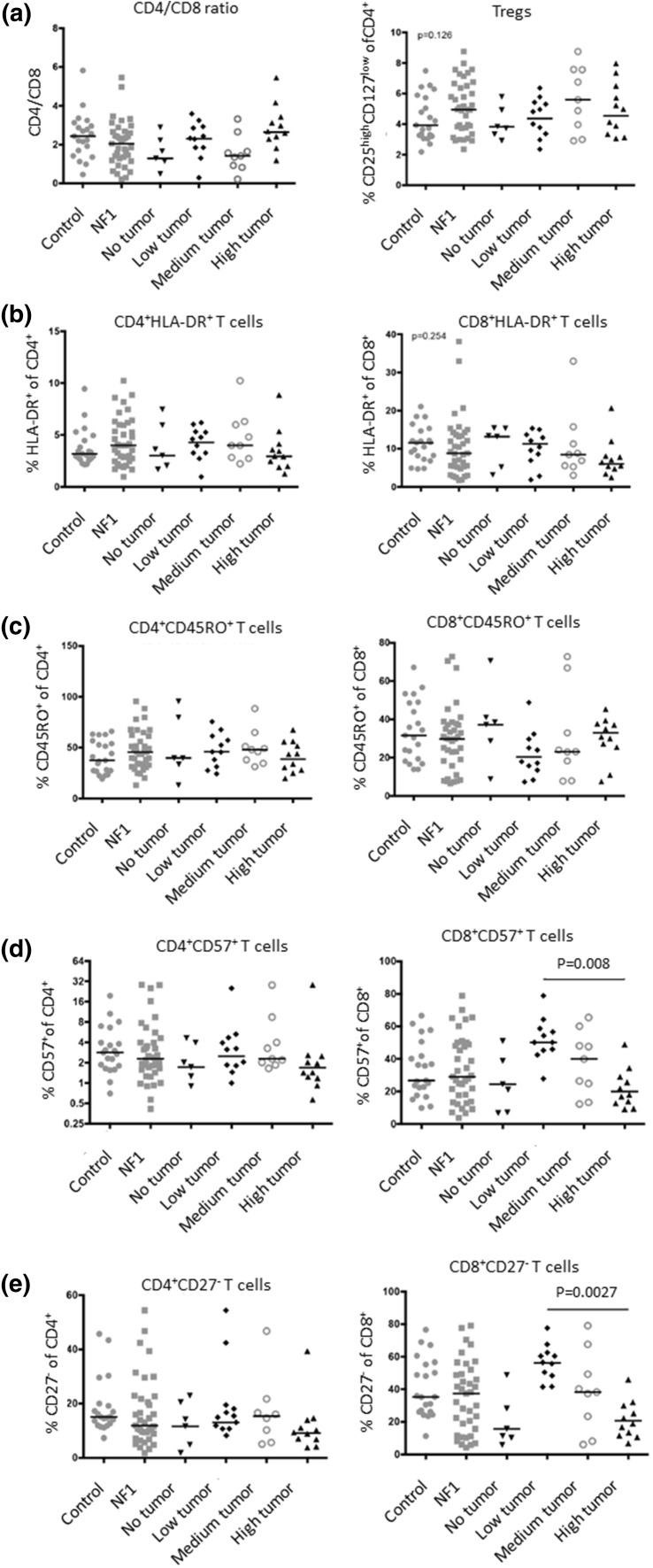
Flow cytometric analysis of the ratio between CD4 and CD8 cells, and the percentages of different T cell populations in PBMC samples of healthy age- and gender-matched controls. **a** CD4/CD8 ratio and regulatory T cells as defined by CD4^+^CD25^high^CD127^low^ phenotype; **b** frequencies of activated HLA-DR^+^ CD4^+^ and CD8^+^ cells; **c** percentages of CD45RO^+^ memory CD4^+^ and CD8^+^ T cells; **d** percentages of chronically activated CD57^+^ CD4^+^ and CD8^+^ T cells and **e** percentages of CD27^−^ effector CD4^+^ and CD8^+^ T cells

Global blood cell analysis showed a generalized low count of leukocytes in NF1 patients, independent of age. Granulocytes and monocytes are at the lower end of the normal range, while lymphocytes are below the standard reference range (Fig. 1). Our further analysis focused therefore on analyzing T cell subpopulations by flow cytometry of PBMC from NF1 patients over 18 years (*n* = 37) and healthy age-matched controls (*n* = 21) (Table 1). As we aimed at identifying correlations between the immune phenotype and the cancer phenotype, patients were analyzed by whole-body MRI to quantify their total internal tumor load. Children and NF1 patients below 20 years of age were excluded since the tumor phenotype is often not fully established at younger ages. No significant differences were observed between the overall cohort of NF1 patients and the non-NF1 controls for any of the T cell subpopulations (Fig. 2). However, we identified a trend for higher levels of regulatory T cells (Fig. 2a), fewer CD8^+^/HLA-DR^+^ T cells (Fig. 2b) as well as fewer CD4^+^CD27^−^ effector T cells (Fig. 2e) in the general NF1 population. Although these trends were not significant (*p* = 0.06), the analysis clearly revealed groups of patients with very few memory and effector T cells (Fig. 2d, e).

**Table 1:** Phenotype of NF1 patients with quantified dermal and internal tumors

			Number of dermal neurofibroma		Internal tumor (PNF) (volume in cm^3^)				
Patient no	Sex	Age	Subcutaneous	Cutaneous	None*	Low 1–99 cm^3^	Medium 100–500 cm^3^	High >500 cm^3^	MPNST
1	M	22	1	11–50	x				
2	F	40	3	1–10	x				
3	M	25	9	0	x				
4	F	38	0	101–500	x				
5	F	48	11–50	1000+	x				
6	F	26	0	11–50	x				
7	F	45	51–100	101–500		81			
8	F	66	15	11–50		22			
9	M	48	0	11–50		39			
10	M	44	0	1000+		28			
11	F	55	11–50	101–500		44			
12	M	41	0	11–50		16			
13	M	42	0	11–50		22			
14	M	45	0	51–100		74			
15	M	21	0	11–50		38			
16	M	27	11–50	11–50		61			
17	F	42	0	1000+		76			
18	F	53	0	1000+			341		
19	M	23	2	500+			119		
20	M	25	0	11–50			476		
21	F	36	11–50	51–100			326		x
22	F	49	11–50	51–100			382		
23	F	26	0	11–50			491		
24	M	31	0	11–50			212		
25	M	47	11–50	1000+			115		
26	F	35	8	11–50			112		
27	M	26	1000+	1000+				739	x
28	F	54	0	1000+				900	
29	F	24	0	0				810	
30	M	31	10–500	7				5408	
31	F	20	1000+	11–50				950	
32	F	50	51–100	1000+				1191	
33	F	34	0	101–500				508	
34	M	41	9	1000+				577	
35	M	43	0	8				661	
36	F	62	11–50	51–100				1005	
37	M	49	0	51–100				767	

* Volume below detection level (1 cm^3^)

Next, we divided the NF1 patients (*n* = 37) into four different groups according to the total internal PNF tumor volume as determined by whole-body MRI: The groups show no internal tumors (*n* = 5), low tumor volume (*n* = 11), medium tumor volume (*n* = 9) and high tumor load (*n* = 11) and associated the frequency of the T cell subpopulations with these groups (Fig. 2). We found no significant differences between NF1 patients and healthy controls or between NF1 patients with different tumor loads for any of the CD16/CD56 NK cell populations investigated (data not shown). In addition, no associations were found between regulatory T cells and the CD4^+^/HLA-DR^+^, CD45RO^+^, CD57^+^, and CD27^−^ cell subpopulations, as well as the CD8^+^/HLA-DR^+^ and CD45RO^+^ T cell subpopulations (Fig. 2a–e). In contrast, the CD8^+^/CD57^+^ and CD8^+^/CD27^−^ memory and effector T cell populations were significantly and inversely correlated with increased internal tumor burden of NF1 patients (Fig. 2d, e). The levels dropped even below baseline in the NF1 group with the highest tumor load. The association only correlated with internal tumor burden and was independent of age (data not shown). No correlation was found between lymphocyte subpopulations and subcutaneous or cutaneous neurofibromas (data not shown). Although a correlation between the growth rate of plexiform and subcutaneous, but not cutaneous tumors has been described (27), the data may indicate a different role of lymphocytes in internal, subcutaneous and cutaneous tumors as compared to PNFs. Other NF1-related phenotypes were not quantified in the observed healthy and NF1 cohorts, and hence, it was not possible to assess associations with vascular, skeletal or cognitive disease manifestations.

The results indicate that although not significantly different, there is, similar to the significantly different D57^+^ cells, a tendency to also lower frequency of CD8^+^/HLA-DR^+^-activated T cells with increased tumor burden (Fig. 2b). However, for CD8^+^/HLA-DR^+^-activated T- cells, we did not observe an increase from samples of patients having no or low tumor volume as observed for CD57^+^ and CD27^−^ cells (Fig. 2b, d, e). This might be due to different expression kinetics of the respective maturity stages. Interestingly, we did not observe differences in CD45RO^+^ memory T cell frequencies in relation to tumor burden (Fig. 2c). Thus, we only observe significant differences in frequencies of more terminally differentiated (CD57^+^/CD27^−^) T cells, which show the highest functional capacity, indicating an increasing deficit in T cell differentiation with tumor progression.

It is interesting that CD8^+^CD27^−^ T cells in NF1 patients without tumor are lower than in the low tumor group. However, there is a striking increase in frequencies of CD8^+^CD27^−^ T cells from patients with no tumor to low tumor, whereas it declines with further tumor progression (Fig. 2d, e). We hypothesize that at the beginning of tumor formation T cell activation and full differentiation are still intact and that the frequencies increase in order to control the developing tumor. However, during further tumor development and at larger tumor volumes, full T cell differentiation is inhibited by so far undefined mechanisms requiring further investigations in a larger investigation.

**Fig. 3:**
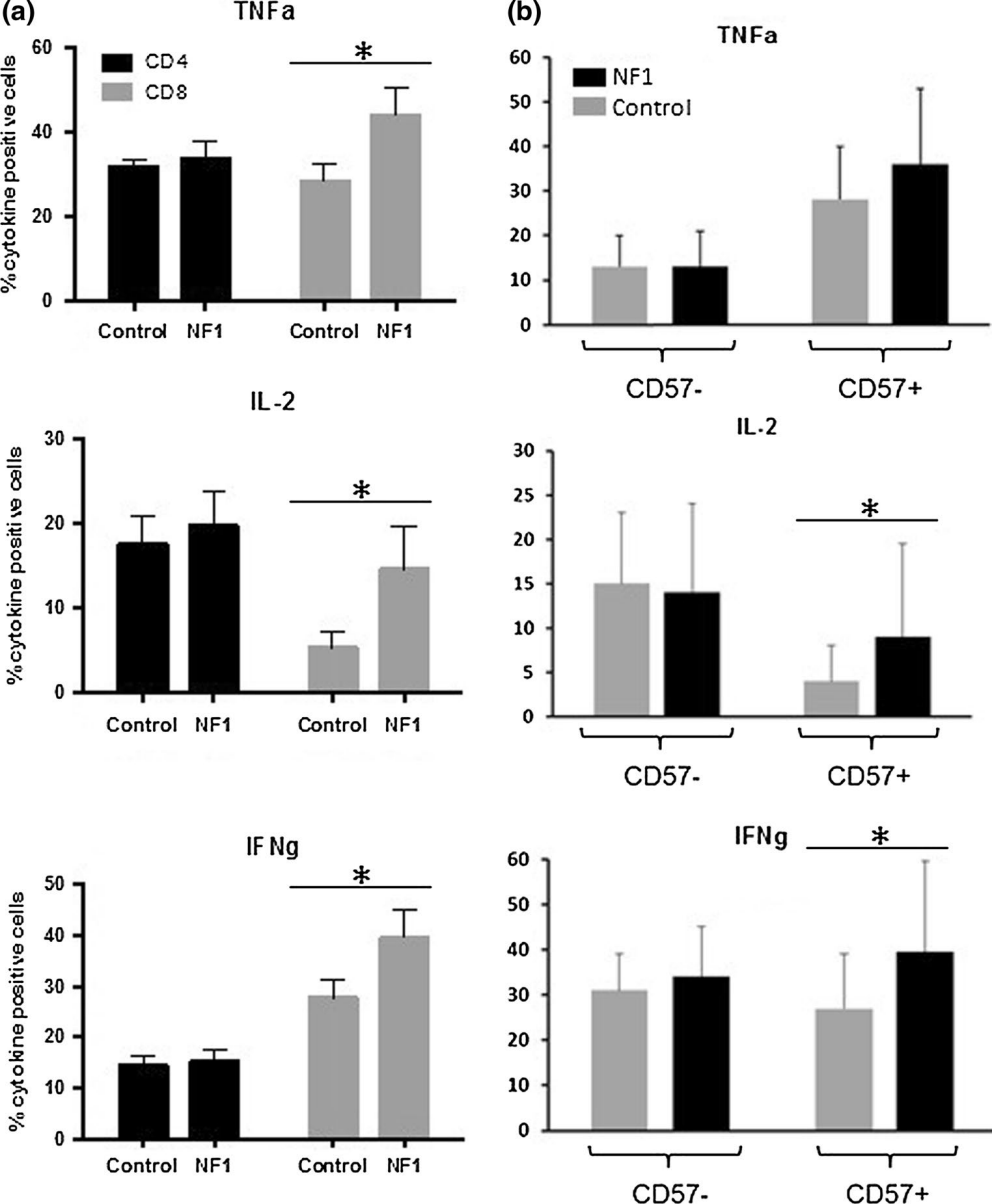
Intracellular cytokine staining after PMA stimulation by CD4 and CD8 cells from age- and gender-matched NF1 patients (*n* = 15) and healthy controls (*n* = 13). IL2, TNF-α and IFN-γ production was determined by FACS (*p < 0.05). **a** Comparison of CD4^+^ and CD8^+^ fractions; **b** comparison of CD8^+^/CD57^−^ and CD8^+^/CD57^+^ fractions

CD57 expression is a characteristic of functional immune deficiency and replicative senescence [26]. We therefore analyzed the capacity of the CD4^+^ and CD8^+^/CD57^+^ T cells in NF1 patients to secrete inflammatory cytokines upon stimulation. Interestingly, IL-2 and IFN-γ production is significantly increased in CD8^+^/CD57^+^ T cells of NF1 patients when compared to healthy controls (*p* < 0.05) (Fig. 3). No difference was found for CD4^+^/CD57^+^ helper T cells. These data suggest that CD8^+^/CD57^+^ replicative exhausted memory T cells in NF1 patients react strongly to stimulation and produce pro-inflammatory cytokines at higher levels than control subjects. There was no correlation between tumor load and cytokine secretion (data not shown), which is in agreement with our previous finding of increased IFN-γ and TNF-α serum levels in NF1 patients independently of tumor load [23].

NF1 patients with high internal tumor load are at high risk for increased morbidity and for development of malignant peripheral nerve sheath tumors (MPNST). The data suggest that the level of CD8^+^/CD57^+^ and CD27^−^ T cells present a useful marker for the internal tumor burden and subsequently the risk to transformation into MPNST. Its clinical use as such a biomarker alone or together with other biomarkers such as insulin-like growth factor binding protein 1 (IGFBP1) [23, 24] will need validation in larger cohorts.

Our findings potentially also indicate that the T cell-mediated immune response in patients with low tumor load is potentially stronger than in patients with high tumor load. Although it would be necessary to substantiate this assumption in a larger cohort, it would suggest that patients with high tumor load only mount a weak cellular immune response, favoring the establishment of a tumor-promoting environment. The data do not distinguish between the alternative hypotheses that aberrant immune cell counts are the consequence rather than a cause of the variable systemic tumor phenotype in NF1 patients. For example, a large tumor burden might increasingly exhaust the existing effector CD57^+^ T cell reservoir. Interestingly, an increased number of CD8^+^CD57^+^ T cells in NF1 patients was also shown in another study [16], although our data confirm such an increase only for the NF1 subgroups with low or medium internal tumor load. It is of note that the CD8^+^/CD27^−^ populations in the NF1 group without internal tumors were smaller than in any other subgroup, including the healthy control group. The aberrantly low levels of this T cell population were thus not dependent on tumors and caused by the tumor phenotype. Although the group size is too small for statistically useful evaluation, this trend may suggest a general difference in the T cell phenotype of NF1 patients.

In conclusion, our results show that the CD8 memory and effector T cell levels are inversely correlated with tumor burden in NF1 patients. Hence, the ratios of CD8^+^/CD57^+^ and CD27^−^ T cells may present a useful surrogate marker for the internal tumor burden of NF1 patients, which justifies further validation in a larger cohort.

## Abbreviations

APC: Allophycocyanin
CD: Cluster of differentiation
DMSO: Dimethylsulfoxide
ECD: Electron coupled dye
FACS: Fluorescence-activated cell sorting
FBS: Fetal bovine serum
FITC: Fluorescein isothiocyanate
GAP: GTP activating domain
GTP: Guanosine triphosphate
HLA: Human histocompatibility leukocyte antigen
HLA-DR: HLA antigen D related
IFN: Interferon
IL: Interleukin
MRI: Magnetic resonance imaging
NF1: Neurofibromatosis type 1
*NF1/Nf1*: Neurofibromin gene (human/mouse)
PBMC: Peripheral blood mononuclear cells
PBS: Phosphate-buffered saline
PE: Phycoerythrin
PE-Cy: PE-cyanin
PerCP: Peridinin chlorophyll protein
PMA: Phorbol myristate acetate
PNF: Plexiform neurofibroma
STIR: Short-Ti inversion-recovery
T cell: Thymocyte
TNF: Tumor necrosis factor
